# Heat-Treated Ni-CNT Nanocomposites Produced by Powder Metallurgy Route

**DOI:** 10.3390/ma14185458

**Published:** 2021-09-21

**Authors:** Íris Carneiro, Sónia Simões

**Affiliations:** 1 DEMM, Department of Metallurgical and Materials Engineering, University of Porto, R. Dr. Roberto Frias, 4200-465 Porto, Portugal; up201207199@fe.up.pt; 2 LAETA/INEGI, Institute of Science and Innovation in Mechanical and Industrial Engineering, R. Dr. Roberto Frias, 4200-465 Porto, Portugal

**Keywords:** nanocomposites, heat treatment, grain growth, carbon nanotubes, powder metallurgy

## Abstract

Nickel nanocomposites reinforced by carbon nanotubes (Ni-CNTs) are one of the possible candidates for applications in highly demanding industries such as the automotive and aerospace industries. As is well known, one of the limitations on the use of some materials in these applications is thermal stability. Some components in these industries are frequently subjected to high temperatures, which is crucial to understanding their microstructures and, consequently, their mechanical properties. For this reason, the main objective of this research is to understand the microstructural evolution of Ni-CNTs nanocomposites when subjected to heat treatment. The nanocomposites with varying levels of CNT content were produced by powder metallurgy, and unreinforced nickel was used for comparison purposes under the same conditions. The dispersion of CNTs, a critical aspect of nanocomposites production, was carried out by ultrasonication, which already proved its efficiency in previous research. The heat treatments were performed under high vacuum conditions at high temperatures (700 and 1100 °C for 30 and 120 min, respectively). Microhardness tests analyzed the mechanical properties while the extensive microstructural evaluation was conducted by combining advanced characterization techniques such as scanning electron microscopy (SEM) with electron backscatter diffraction (EBSD), transmission electron microscopy (TEM), and high-resolution TEM. The obtained results are promising and show that the presence of CNTs can contribute to the thermal stability of the Ni-CNT nanocomposites produced.

## 1. Introduction

Recently, environmental consciousness is increasing along with stricter regulations and the urgent need to reduce fuel consumption. One of the possible ways to achieve this is by reducing the weight of structural components through metal matrix nanocomposites [1,2].

The use of metal matrix nanocomposites shows the great advantage of combining a matrix and reinforcing material properties, producing an advanced material with exceptionally high-level properties tailored for the intended application. However, it is essential to acknowledge that several factors can play a determining role in the properties of the nanocomposite, such as the production route, the reinforcement dispersion efficiency, the interaction between the constituents, and which matrix and reinforcement materials are combined [1,3].

Although there is a wide range of possible candidates available as reinforcements, such as some carbides [4,5,6,7] and oxides [8,9,10,11], carbon nanotubes (CNTs) have been living up to their great potential since their discovery by Iijima [12]. Their unique structure, with a high aspect ratio and large surface area, low density, and excellent properties, makes them a suitable and efficient material to reinforce several metallic matrices [13,14,15,16,17,18]. 

Due to their excellent and unique properties, CNTs’ reinforced nickel nanocomposites are potential materials for applications in highly demanding industries such as in automotive and aerospace components, which are frequently subjected to high temperatures. With this in mind, it is crucial to understand the effect of temperature application over time on their microstructure, since this directly affects the material’s mechanical properties. 

The service temperature can induce grain growth if the material selection is incorrect, compromising its mechanical properties and service performance. The effect of the reinforcement material on thermal stability is an important consideration if the objective is to apply the nanocomposite to a component that is subjected to high temperature. Some published research [19,20,21,22,23] revealed the effects of CNTs on the behavior of the metal matrix during heat treatment. 

Suárez et al. [19] investigated the effects of different amounts of CNTs (1.00 and 3.00% wt.) on heat-treated nickel nanocomposites produced by hot pressing and sintering following torsion to achieve a nanocrystalline structure through severe plastic deformation. The authors performed annealing at ≈300 °C for 180 min on the plastically deformed samples to study their thermal stability. After sintering, the nanocomposites presented a smaller grain size than the pure nickel. The severe deformation process caused a significant decrease in grain size. The authors observed that the nickel grains grew more significantly during the heat treatment than the grains in the nanocomposites, which can be attributed to the grain boundary pinning effect caused by the presence of CNTs.

Aristizabal et al. [20] produced nickel nanocomposites by solid-state processing followed by several plastic deformations. The heat treatment was performed at 500 °C for 12 h. The results revealed that the CNTs influenced the microstructural evolution during the annealing process, improving the thermal stability of the nanocomposites relative to the unreinforced samples, with the average grain size varying according to the CNT content. This can be explained by the fact that CNTs, even with some damage, pin the grain boundaries, which restricts grain growth during annealing.

However, this research relates to composites produced by deformation processes, characterized by microstructures exhibiting high plastic deformation or by using metal alloys as a matrix. There is still a lack of knowledge on the thermal stability of nanocomposites produced only by conventional powder metallurgy processes.

Therefore, the main objective of the present research is to evaluate and understand the microstructural evolution of nickel matrix nanocomposites produced by powder metallurgy route, reinforced with varying CNT content, when subject to different heat treatment temperatures and durations, and mainly, to understand the effects of CNTs on the microstructural evolution of the nanocomposites.

## 2. Materials and Methods

The nanocomposites used in this research were produced by powder metallurgy. Nickel powder was provided by Goodfellow Cambridge Ltd. (Huntingdon, UK) and multi-walled carbon nanotubes (MWCNTs) were obtained from Fibermax Nanocomposites Ltd. (London, UK), which were already characterized in previous research [14,24,25,26,27]. 

The Ni and CNT powders were mixed by ultrasonication and cold-pressed under a pressure of 900 MPa. The sintering was performed under vacuum at 950 °C for 120 min. Different volume fractions (0.50, 0.75, 1.00, 1.50, 1.75 and 2.00 vol.%) of the CNTs were used. Heat treatment of the samples was conducted at 700 °C for 120 min and 1100 °C for 30 and 120 min. Higher temperatures were selected to understand the limitations of the effects of the CNTs on the microstructural evolution of the nanocomposites. 

The microstructural characterization of the nanocomposites was attained by combining different techniques. Optical microscopy (OM) combined with image analysis software was utilized to examine samples and measure the average grain size. A DM 4000 M optical microscope equipped with a Leica DFC 420 camera (Leica Microsystems, Wetzlar, Germany) was used to perform this task while the image analysis quantification was performed with Leica Application Suite software (Leica Microsystems, Wetzlar, Germany). 

The preparation of samples for optical microscopy consisted of a conventional metallography polishing sequence (180, 320, 500 and 1000 mesh, followed by diamond suspensions of 6 and 1 µm, and colloidal silica of 0.06 µm) and an etching with Kroll reagent (4% HNO_3_, 6% H.F., 23% H_2_O_2_ and 67% H_2_O) for 30 s to reveal the grain boundaries. For the EBSD sample preparation, the step with colloidal silica was conducted for 6 h. Electron transparent cross-sections of nanocomposites were prepared by the lift-out technique at 5–30 keV, using a focused ion beam (FIB) (Thermo Fisher Scientific, Hillsboro, OR, USA).

Electron backscatter diffraction (EBSD) in scanning electron microscopy (SEM) was used to characterize different microstructural features in detail. For this characterization, a high-resolution Thermo Fisher Scientific QUANTA 400 FEG SEM (Thermo Fisher Scientific, Hillsboro, OR, USA) coupled with an EBSD detector TSL-EDAX EBSD Unit (EDAX Inc. (Ametek), Mahwah, NJ, USA) was used. EBSD data was analyzed using TSL OIM Analysis 5.2 2007 (EDAX Inc. (Ametek), Mahwah, NJ, USA) and different maps were performed similar to that of previous research [24,25], to obtain detailed information on the microstructures of the samples. Before all the maps were elaborated, all the raw data was subjected to a clean-up process, defining a grain tolerance angle of 15° and a minimum grain size of 2 points. The inverse pole figures (IPF) permitted the understanding of the crystallographic orientation, both in the individual grains and in the overall microstructure, and the possible existence of preferred orientation. Assessing the presence of deformation in Ni and Ni-CNT nanocomposites was carried out by combining Kernel average misorientation (KAM) maps, which is one of the indicated types of maps utilized specifically to study the deformation, and image quality (IQ) maps with the low- and high-angle boundaries defined. The low-angle boundaries are associated with dislocation cells, while the high-angle boundaries are representative of grain boundaries. The influence of CNTs and heat treatment on the recovery and recrystallization processes was evaluated through grain orientation maps (GOS) and recrystallization maps. The recrystallization maps are the preferred ones to study these processes since their combination allows for the analysis of the microstructure evolution and the categorization of the grains (recrystallized, recovery substructure, and deformed). The deformed grains exhibit a high grain average misorientation, while the recrystallized grains exhibit a low grain average misorientation (from 0 to 2°). 

X-ray diffraction (XRD) was used to investigate the eventual second phase formation, using a Panalytical X’Pert Pro MPD diffractometer (Malvern Panalytical Ltd., Malvern, UK) with CuKα radiation and collecting patterns from 20° to 100° (2θ) in a θ–2θ Bragg-Brentano mode.

Differential scanning calorimetry (DSC, Setaram Kep Technologies, Caluire-et-Cuire, France) was performed from room temperature to 750 °C, with a heating rate of 5 °C min^−1^ in an argon atmosphere.

The use of high-resolution transmission electron microscopy (HRTEM) (Thermo Fisher Scientific, Hillsboro, OR, USA) was indispensable in the detailed characterization of the CNTs bonding in the nickel matrix. Due to its reliability and resolution, this technique is essential to analyze the formation of second phase particles resulting from the CNT and metal matrix reactions, especially if there are very few or if the particles are present on a nanometric scale, and in order to evaluate a damaged CNT structure. 

Mechanical characterization was performed by Vickers microhardness tests carried out on a Duramin-1 durometer (Duramin-1; Struers A/S, Ballerup, Denmark), executing the 10 to 15 indentations per sample on its cross-section and with a load of 98 mN applied for 15 s.

## 3. Results and Discussion

**Figure 1:**
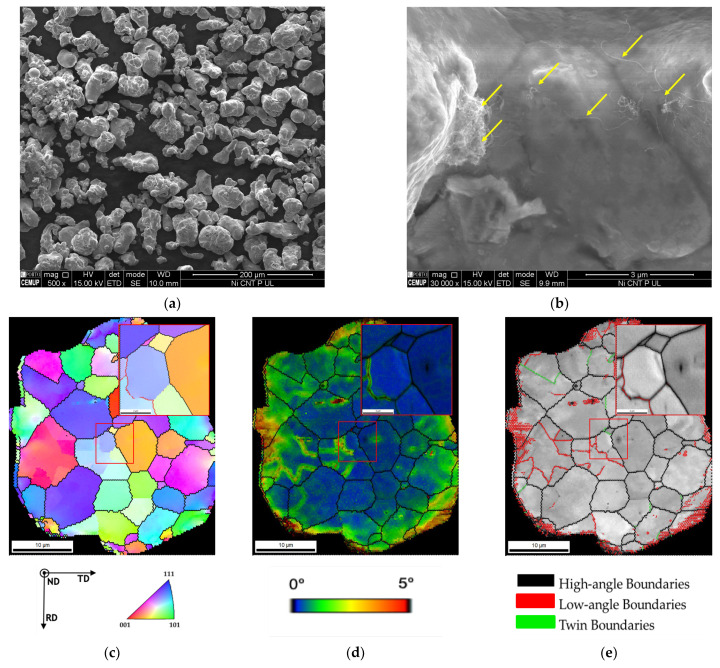
(**a**,**b**) SEM images of Ni-CNT particles after ultrasonication showing the presence of CNTs (yellow arrows) and (**c**) Inverse pole figure (IPF) map, (**d**) Kernel average misorientation (KAM), and (**e**) image quality (IQ) with high- and low-angle boundaries and twin boundaries delineated.

In the powder metallurgy route, the dispersion/mixture is a crucial step of the process for the final sintered microstructure. The effects of this method on the microstructures of the Ni-CNT powders were evaluated by SEM and EBSD results. Figure 1 shows the SEM images and the Inverse pole figure (IPF) map, Kernel average misorientation (KAM), and image quality (IQ) with high- and low-angle boundaries and twin boundaries delineated of a Ni-CNT particle after ultrasonication process. SEM images in Figure 1a reveal that some particle agglomeration occurred during the process, promoting a change in the shape of the Ni powder [25]. Figure 1b shows a higher magnification proving the presence of CNT in the Ni powder. From IPF maps, no preferred crystallographic orientation was detected, indicating that the process did not lead to texture formation. High misorientation and high density of low-angle grain boundaries on the particle’s surface can be confirmed by the KAM and IQ maps. This increase in the density of the grain boundaries is undoubtedly associated with plastic deformation occurring during the process.

**Figure 2:**
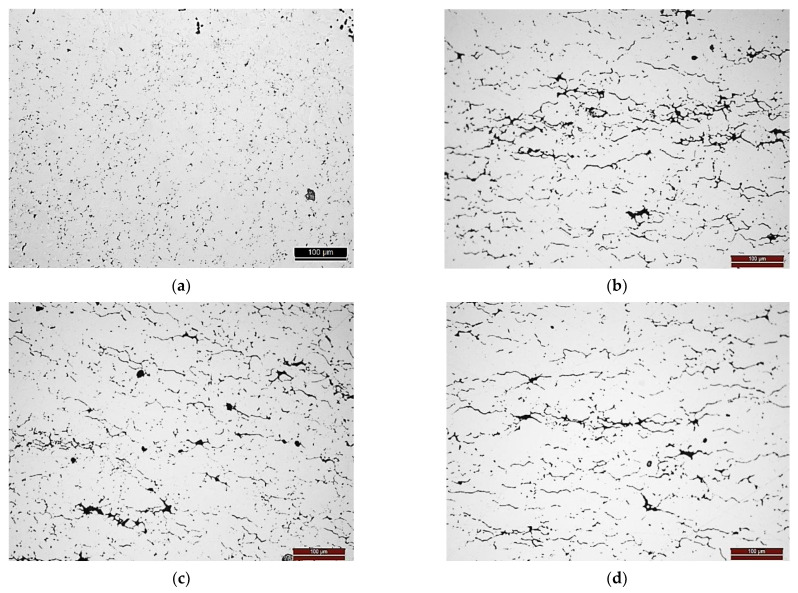
OM images of the samples heat-treated at 1100 °C for 30 min: (**a**) Ni sample, (**b**) Ni-0.50 vol.% CNT, (**c**) Ni-0.75 vol.% CNT and (**d**) Ni-1.00 vol.% CNT.

The heat treatment did not affect the microstructures of the nanocomposites in terms of the CNT clusters distribution. In Figure 2, it is possible to observe the optical microscopy images for the samples heat-treated at 1100 °C for 30 min. As already mentioned in previously published research [24,26,27], nanocomposites exhibit the CNT clusters mainly at the grain boundaries and pores. In the OM images, the pores and CNTs clusters can be identified as the dark phase. The relative densities of the Ni and Ni-CNT samples are 0.824 and 0.785, respectively. 

**Figure 3:**
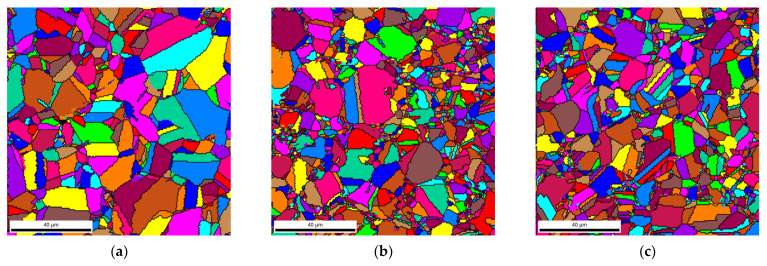
Unique grain color maps of heat-treated samples at 1100 °C for 30 min of (**a**) Ni, (**b**) Ni-0.50 vol.% CNT, and (**c**) Ni-1.00 vol.% CNT.

**Figure 4:**
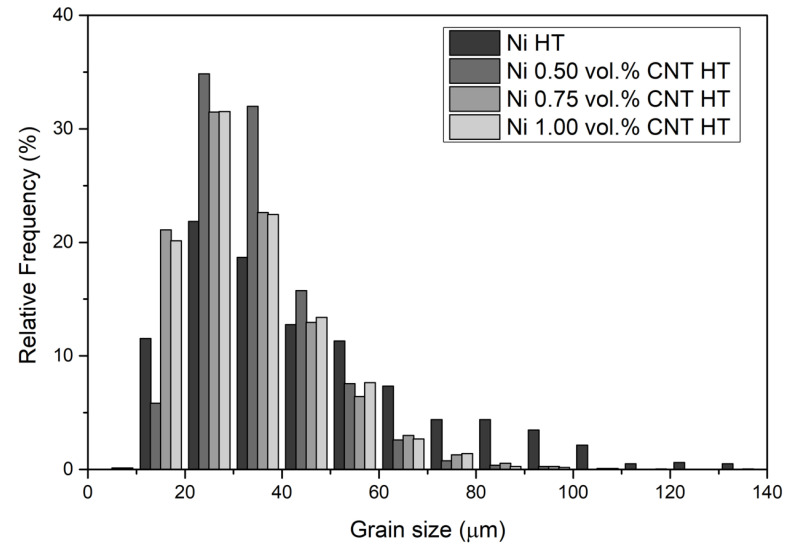
Grain size distributions of Ni and nanocomposites produced with 0.50, 0.75, and 1.00 vol.% of CNT heat-treated (HT) at 1100 °C for 30 min.

Unique grain color maps and grain size distributions of the Ni and nanocomposites can be observed in Figure 3 and Figure 4 for the Ni and the nanocomposites heat-treated at 1100 °C for 30 min. Based on the grain size distributions, it is evident that the nanocomposites exhibited a smaller average grain size. The nanocomposites revealed more grains with smaller grain sizes than the Ni samples. These results can be attributed to the fact that the CNT clusters can act as obstacles to the movement of the grain boundaries during the increase in temperature, preventing grain growth. This effect will be more pronounced the better the CNTs are dispersed in the matrix. 

The unique grain color maps of Figure 3 clearly show the effects of the CNTs on grain growth during the heat treatment of the samples. The average grain size for the nanocomposites is smaller than that of the Ni samples heat-treated under the same conditions. Near the CNT clusters, the grains are smaller as CNTs hinder grain growth. To obtain a practical grain size reduction effect throughout the nanocomposite, uniform dispersion of the CNTs is necessary. As discussed in previous research, such a condition is observed for nanocomposites with 1.00 vol.% of CNT [14,26]. When larger CNT clusters are present in the matrix, abnormal grain growth is visible; near the clusters, grain growth is minimal, and whole grains that have neither cluster grow to sizes close to those observed in nickel matrices.

**Figure 5:**
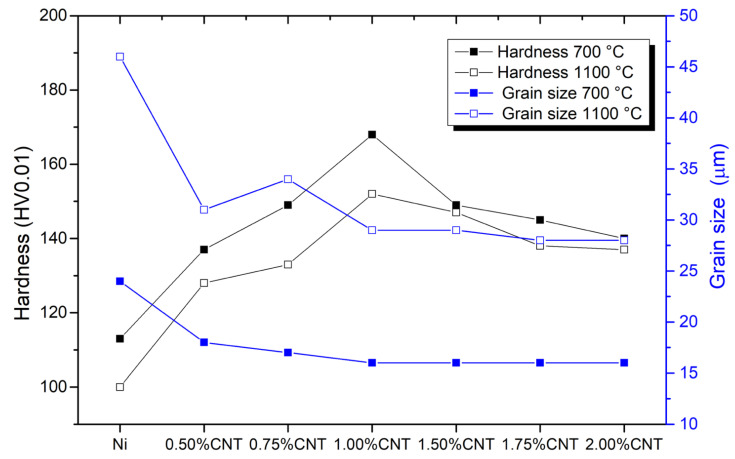
Hardness (HV0.01) and grain size (µm) evolution for Ni and nanocomposites heat-treated at 700 °C for 120 min and 1100 °C for 30 min.

In Figure 5, the evolution of grain size and hardness of both Ni and nanocomposites produced with different amounts of CNT heat-treated at 700 and 1100 °C can be seen. Based on this figure, it is possible to observe that the heat treatments increased the grain size and decreased the hardness. With the rising heat treatment temperature, the increase of the grain size is evident for the Ni matrix. However, the presence of the CNT in the Ni matrix affects grain growth. For the nanocomposites, the grain size increase is smaller than for the Ni sample, and consequently, the hardness values are lower. Increasing the amount of CNT led to a stabilization of the values, although, for 1.00 vol.% CNT had a smaller grain size and a higher hardness value. This can be attributed to the fact that for this amount of CNT, a better dispersion of the CNTs can be observed [14,26]. 

**Figure 6:**
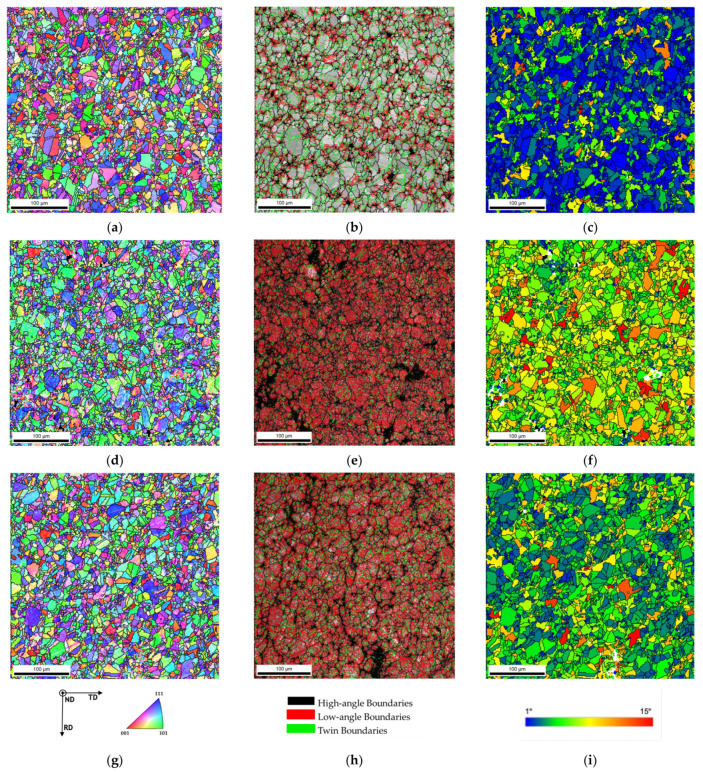
Inverse pole figure (IPF) map, image quality (IQ) with high- and low-angle boundaries and twin boundaries delineated, and grain orientation spread (GOS) maps of (**a**–**c**) Ni heat-treated at 700 °C for 120 min, (**d**–**f**) Ni-1.00 vol.% CNT heat-treated at 700 °C for 120 min, and (**g**–**i**) Ni-1.00 vol.% CNT heat-treated at 1100 °C for 30 min.

Besides the grain size, other microstructural characteristics are clearly different between the nickel matrix and the nanocomposites when subjected to heat treatment. Figure 6 shows the inverse pole figure (IPF) maps, image quality (IQ) with high- and low-angle boundaries delineated, and grain orientation spread (GOS) maps of Ni heat-treated at 700 °C and Ni-1.00 vol.% CNT heat-treated at 700 and 1100 °C. The results presented in this figure reveal that the nanocomposites exhibit different grain orientations by IPF maps, a higher density of low-angle grain boundaries (LAGB), and a lower density of recrystallized grains (observed by the GOS maps) than the Ni matrix. The presence of CNTs has a significant effect on the microstructures of the heat-treated nanocomposites.

**Figure 7:**
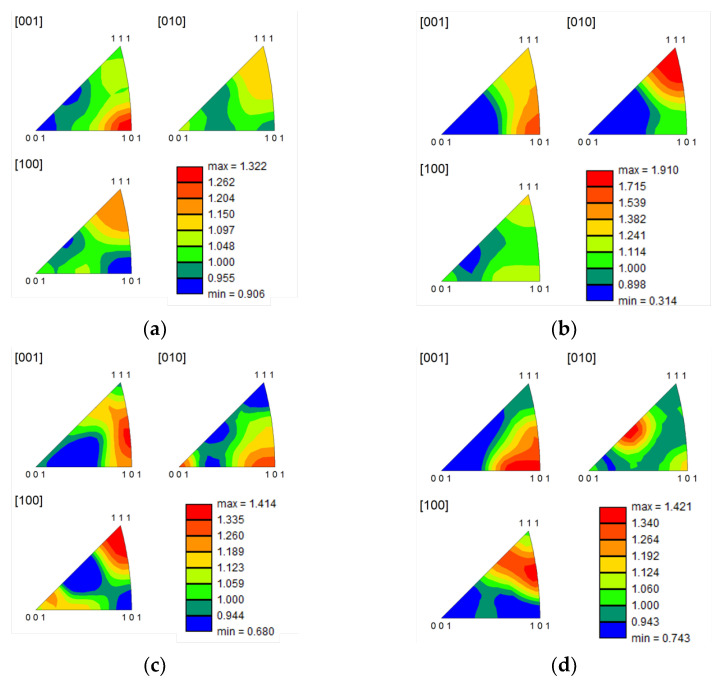
Inverse pole figure (IPF) of (**a**) Ni heat-treated at 700 °C for 120 min, (**b**) Ni-1.00 vol.% CNT heat-treated at 700 °C for 120 min, (**c**) Ni heat-treated at 1100 °C for 30 min and (**d**) Ni-1.00 vol.% CNT heat-treated at 1100 °C for 30 min.

IPF maps indicated that the nanocomposites revealed more grains corresponding to the (101) planes (green grains) on the heat-treated samples than the Ni matrix. This means that the crystalline orientation for the nanocomposites is different, so the CNTs affect the grain rotation during the heat treatment. Figure 7 shows the inverse pole figure graphs for the samples with and without CNT heat-treated at 700 and 1100 °C. These images confirm that the preferential crystal orientation for the nanocomposites is different than for the Ni matrix.

**Figure 8:**
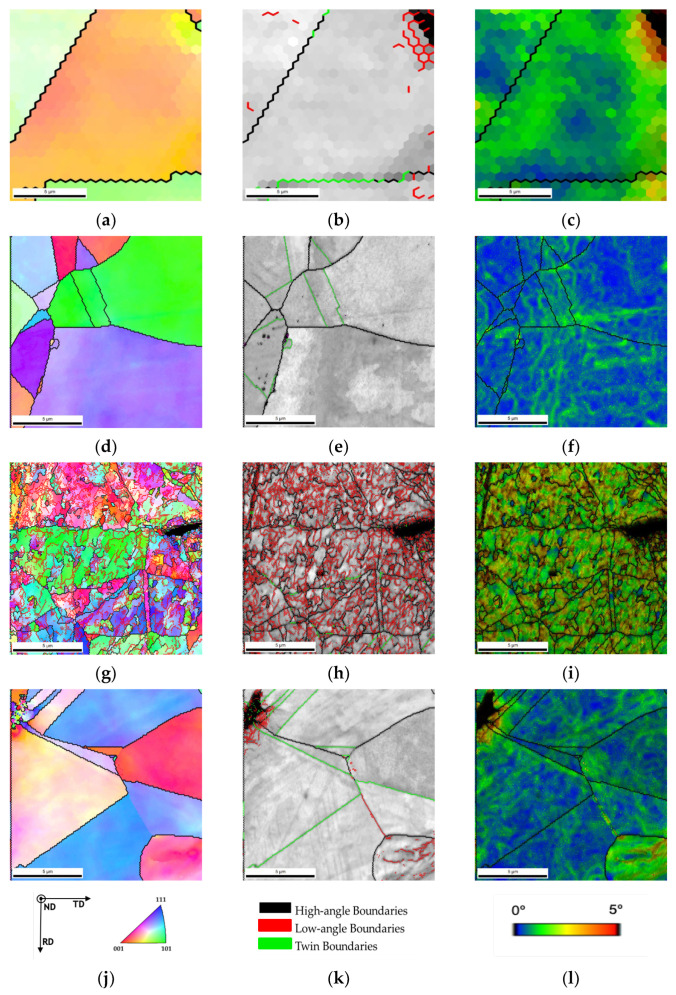
IPF, IQ with high- and low-angle boundaries and twin boundaries delineated, and Kernel average misorientation (KAM) maps of (**a**–**c**) Ni heat-treated at 700 °C for 120 min (**d**–**f**) Ni heat-treated at 1100 °C for 30 min, (**g**–**i**) Ni-1.00 vol.% CNT heat-treated at 700 °C for 120 min and (**j**–**l**) Ni-1.00 vol.% CNT heat-treated at 1100 °C for 30 min.

Another essential difference observed in Figure 6 is the density of the low-angle grain boundaries (LAGBs). For the nanocomposite, LAGB density is higher. Previous research [24,25,28] has already shown that after sintering, nanocomposites reveal more LAGB than the metal without reinforcement due to the CNTs’ influence in increasing the dislocation density. It was expected that with the heat treatments, LAGB density would decrease. However, for the nanocomposite samples treated at 700 °C, a high density of LAGBs was visible, meaning that the CNTs maintained the effect of hindering dislocation motion during the heat treatment and prevented its rearrangement. In the case of Ni, the dislocations that had not been eliminated or rearranged during sintering occurred during the heat treatment. For nanocomposites heat-treated at 1100 °C, there is a slight decrease in the density of LAGBs. This means that even at 1100 °C for 30 min the CNTs interacted with the dislocation motion and rearrangement. Figure 8 shows high-magnification IPF, IQ and KAM. Although the nanocomposites heat-treated at 1100 °C exhibit a low density of LAGBs the KAM maps show more misorientation than in the Ni matrix implying a higher dislocation density, which may be associated with CNT embedded in the matrix.

During the heat treatment, the recovery and recrystallization processes occurred with the rearrangement and annihilation of dislocations due to the plastic deformation of the production process. The LAGBs can be associated with the dislocation cells and the higher density observed for the nanocomposites due to the dislocation interaction with the CNTs. The CNTs act as obstacles to the dislocation motion, hindering the recovery processes. GOS and recrystallization maps were used to investigate the effects of the CNTs in these processes during the heat treatments.

**Figure 9:**
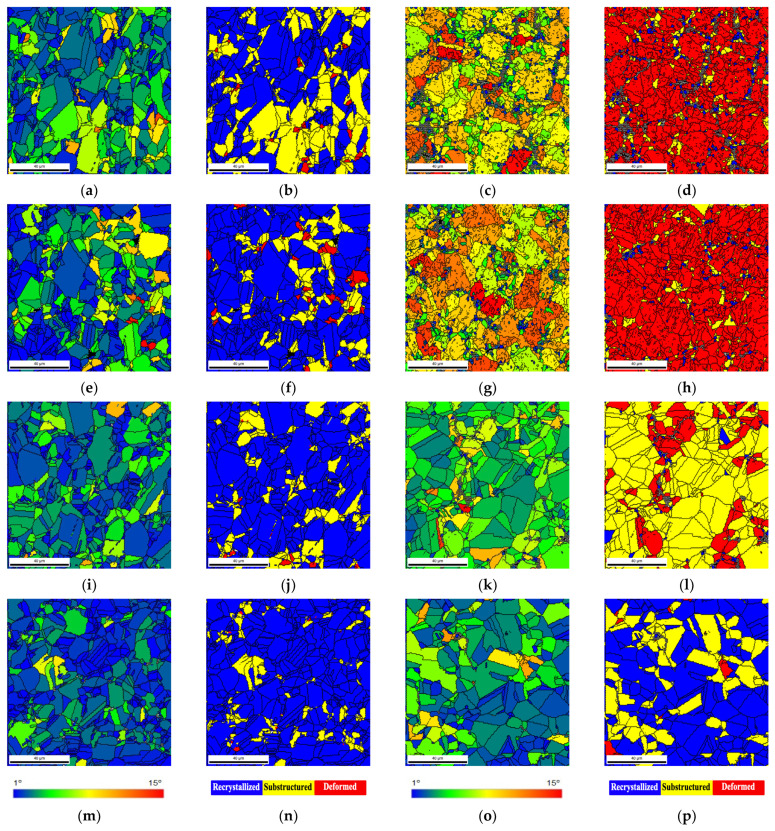
GOS and recrystallization maps of (**a**,**b**) Ni as-sintered, (**c**,**d**) Ni-1.00 vol.% CNT as-sintered, (**e**,**f**) Ni heat-treated at 700 °C for 120 min, (**g**,**h**) Ni-1.00 vol.% CNT heat-treated at 700 °C for 120 min, (**i**,**j**) Ni heat-treated at 1100 °C for 30 min, (**k**,**l**) Ni-1.00 vol.% CNT heat-treated at 1100 °C for 30 min, (**m**,**n**) Ni heat-treated at 1100 °C for 120 min, (**o**,**p**) Ni-1.00 vol.% CNT heat-treated at 1100 °C for 120 min.

The GOS maps of Figure 6 revealed that the nanocomposites show lower blue grains, corresponding to values up until 1° of orientation spread, representing the recrystallized grains. This means that a lower density of recrystallized grains is formed during the heat treatments. On the other hand, the Ni sample heat-treated at 700 °C exhibits a high density of blue grains. Figure 9 shows a higher magnification GOS and recrystallization maps for the heat-treated Ni and nanocomposite samples.

Based on these images, the nanocomposites are characterized by deformed grains, for the as-sintered and heat-treated at 700 °C. For the nanocomposite heat-treated at 1100 °C for 30 min, a high density of recovery substructure grains is observed. Only an increase of the time at 1100 °C promotes the formation of recrystallized grains. For Ni samples, some recrystallization is observed during the sintering, as already reported [24,25,28]. The heat treatment promotes an increase in Ni recrystallization of the grains. Nanocomposites only exhibit a microstructure like the nickel matrix under demanding thermal treatment conditions (e.g., high temperature 1100 °C for 120 min). Under these conditions, damage to the CNTs already compromises its reinforcement and interaction with dislocation and grain boundaries motion. However, more recrystallized grains and twin boundaries are observed in the Ni matrix.

Damage to the structure of the CNTs and the reactions between the matrix and the CNTs are essential aspects to consider as they can also affect the thermal stability of the nanocomposites. Regarding the damage to the structure of CNTs, the inhibition of grain growth can be affected as the structure of the CNT is impaired, which can lead to the elimination of the anchoring process of the grain boundaries during the heat treatment. The chemical reaction between the matrix and the CNTs is another critical parameter to be evaluated since the heat treatment can induce a potent reaction and the formation of carbides. Carbides may also play a role in the thermal stability of nanocomposites.

**Figure 10:**
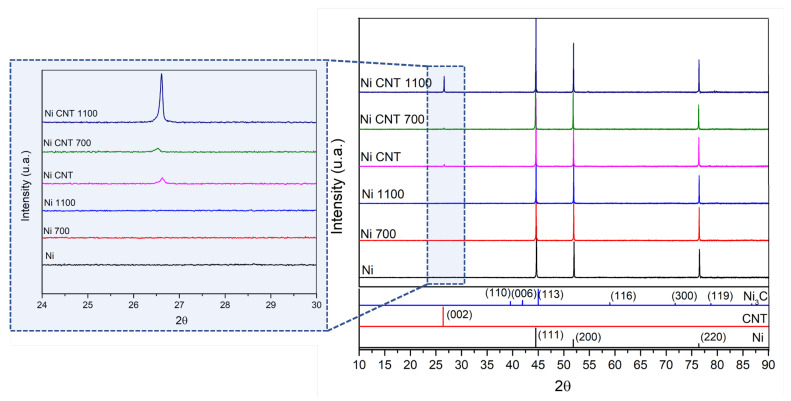
XRD patterns of Ni and Ni-CNT nanocomposite as-sintered and heat-treated at 700 °C for 120 min and 1100 °C for 30 min and XRD peaks for Ni, C, and Ni_3_C phases.

**Figure 11:**
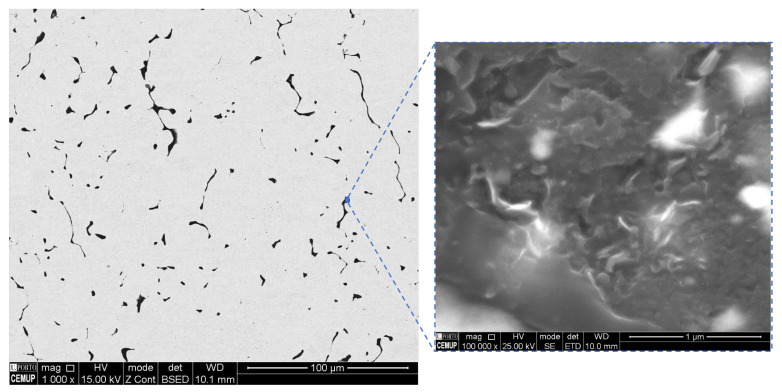
SEM images of Ni-CNT nanocomposite as-sintered and heat-treated at 1100 °C for 30 min showing high magnification images with the CNTs damage.

Figure 10 shows the results of XRD and as-sintered samples, and heat-treated Ni and Ni-CNT samples. The formation of oxides was not detected. It is important to note that sintering and heat treatment were conducted under vacuum better than 10^−2^ bar. For the nanocomposites, (002) carbon planes are visible, which the presence of the CNTs can explain. For the nanocomposite heat-treated at 1100 °C, the carbon peak’s intensity is higher, which means that graphitization occurred (formation of graphene layers which undergo structural reordering due to the damage of the CNT structure) [29]. SEM images of the nanocomposite heat-treated at 1100 °C are present in Figure 11, showing that the CNT structure suffered damage for a longer period of time at higher temperatures than other treatments. The XRD analysis did not detect the Ni_3_C phase. In fact, for the sintered Ni-CNT samples, in previous research [24], the XRD patterns did not detect Ni_3_C, which is formed in nanometric particles. This could mean that the heat treatments did not promote an extensive reaction of the CNT into the carbide phase and, therefore, it is not an essential factor for the thermal stability of the nanocomposites.

**Figure 12:**
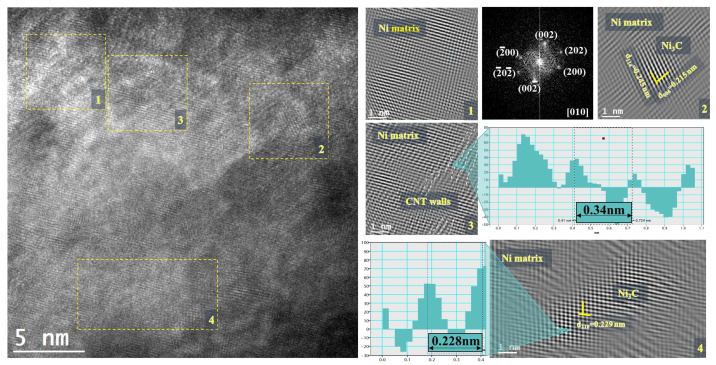
HRTEM image of a Ni-CNT nanocomposite heat-treated at 700 °C with different marked regions where inverse Fast Fourier Transform (FFT) was performed showing the presence of CNT and Ni_3_C phases in the matrix.

The damage to the structure of CNTs and the formation of carbides were analyzed by TEM and HRTEM. In Figure 12, it is possible to observe the HRTEM image for the Ni-CNT heat-treated at 700 °C. Different regions marked in this image were analyzed to detect the formation of different phases and the presence of the CNT in the matrix. Fast Fourier Transform (FFT) and inverse FFT showed that, in this region, the Ni matrix has a (010) zone axis. It is possible to observe some CNT (region 3) walls in the Ni matrix. However, Ni_3_C is visible in two nanoparticles in this HRTEM image (regions 2 and 4). 

The TEM and HRTEM analyses show the CNTs embedded in the matrix as a structure without apparent damage, which is beneficial for acting as an obstacle to the movement of grain boundaries. In addition to the structure of CNTs, it was possible to observe some nanometric particles of Ni_3_C. These particles show the reaction between the matrix and the CNT in very localized zones at 700 °C.

**Figure 13:**
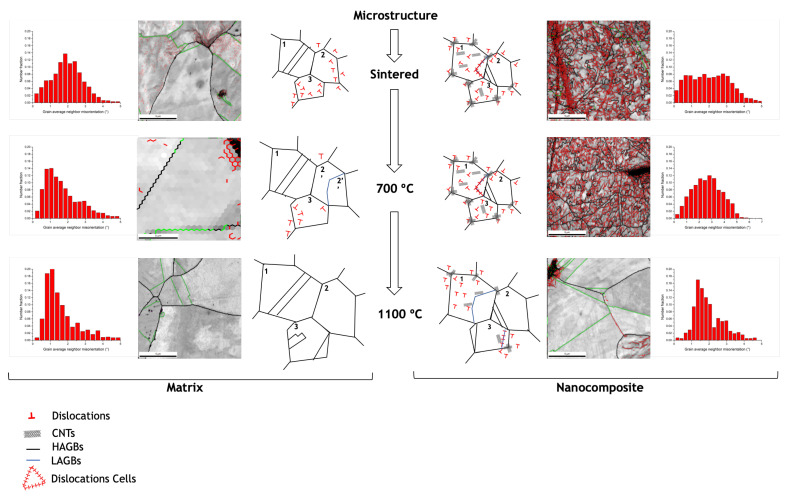
Grain average neighbor misorientation distribution; high magnification images present the microstructures with HAGBs, LAGBs and twins marked, and schematic representation of the matrix and nanocomposites showing the effect of the CNTs on the microstructural evolution of the nanocomposites during heat treatments (HAGBs and LAGBs: high- and low-angle grain boundaries, respectively).

Based on the results, it is possible to present a viable mechanism about the effects of the CNTs on the Ni metal matrix during heat treatments. Figure 13 shows a suggested microstructural evolution for Ni and Ni-CNT during the heat treatments, the images with the different grain boundaries marked, and the grain average neighbor misorientation distribution for the sintering and heat-treated samples.

**Figure 14:**
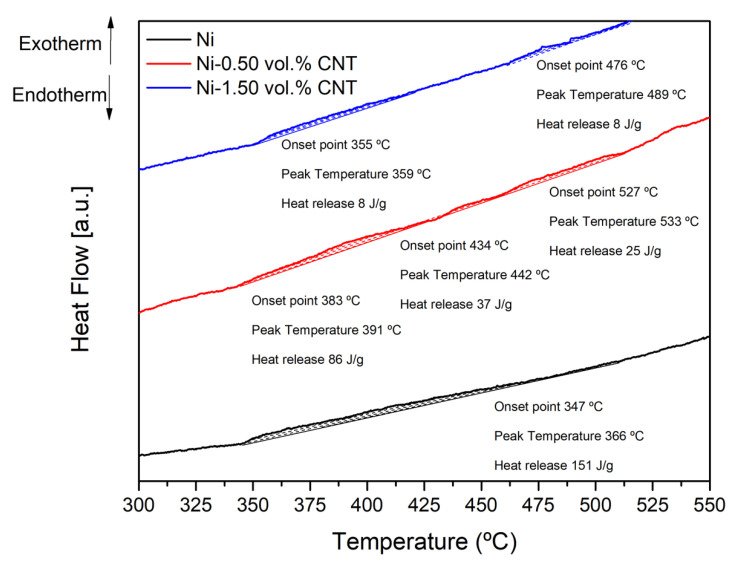
DSC curves of Ni, Ni-0.50 vol.% CNT and Ni-1.50 vol.% CNT nanocomposites.

As already demonstrated in previous research, the presence of CNTs affects the final microstructure of nanocomposites [24]. During sintering, the dislocations formed in the dispersion/mixing processes are reorganized into LAGBs with a ≤5° disorientation (forming subgrains), where some of them are annihilated. At this stage, nanocomposites exhibit high dislocation density close to CNTs, since their presence affects rearrangement and annihilation. Therefore, the sintered microstructure of Ni is composed mainly of recrystallized grains (Grain 1), while the nanocomposites have dislocation cells (Grain 1), dislocation tangles (Grain 3), and the recrystallized grains are of a smaller size (Grain 2) mainly in the vicinity of the CNTs. During heat treatment, grain growth occurs by grain boundary motion (Grain 1) or by coalescence of subgrains for the Ni matrix (Grain 2). The misorientation evolution is affected for the nanocomposite, and the transformation of the grain boundaries from low to high angles is reduced. With the application of temperature over time, the Ni grains grow, and twins are formed, although, for the nanocomposite the presence of CNTs acts as an obstacle and hinders this process. At 700 °C for the Ni matrix, it is possible to observe a reduction in misorientation of the grains and a decrease in the LAGBs, while for the nanocomposites, although the microstructure is very similar to the sintered one, the CNTs has a significant effect on recovery and recrystallization. At 1100 °C for the Ni matrix, the grain growth is more significant, and the increase in the annealing twins is apparent (Grain 3). For the nanocomposites, the microstructure is more uniform than that of the matrix, and subsequently, the damage of the CNTs impairs their effects on the grain boundaries and the dislocation motion. The transformation of LAGBs into HAGBs is evident. The misorientation in the grains decreases due to the dislocation motion from inside the grain, and the grain growth is not inhibited. However, it is still possible to observe some dislocation and LAGBs close to the CNTs clusters and even in some grains that contain some CNTs inside. Since the mobility of the grain boundaries, the extent of the dislocation rearrangement, the coalescence of subgrains, and the misorientation are different in the Ni matrix and the nanocomposites, the evolution of the microstructure will also be different, leading to a different crystalline orientation. Recovery and recrystallization will also be affected depending on the CNT volume fraction. The DSC curve shown in Figure 14 displays this effect on the extent of recrystallization of the nanocomposites. 

**Figure 15:**
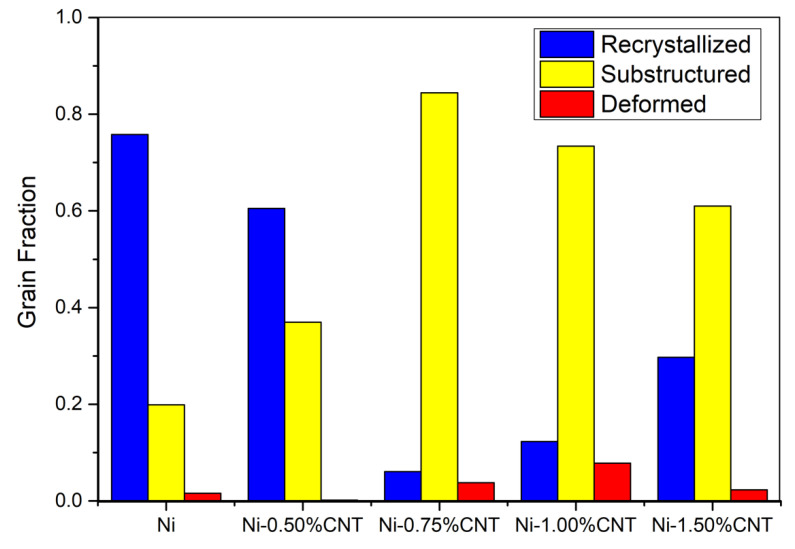
Distribution of recrystallized grains for Ni and nanocomposites with different volume fractions.

An exothermic peak with an onset temperature of 347 °C and a heat release of 151 J/g can be detected for the Ni sample. This exothermic reaction corresponds to the recovery and recrystallization of the sample. For the nanocomposites, it is observed that this reaction occurs in different steps and with a lower heat release. The increase of the volume fraction of the CNTs changes the onset point of the reaction and the amount of heat released. These results show that the introduction of CNTs affects the recrystallization process. This effect is quite evident when observing the distribution of the fraction of recrystallized grains, as shown in Figure 15. It can be observed that Ni has the most considerable fraction of recrystallized grains while nanocomposites have a more significant fraction of substructure recovery and deformed grains.

## 4. Conclusions

The industrial implementation of nanocomposites may be subject to the thermal stability of these materials since the potential applications of these materials require high service temperatures. It is essential to understand to what extent the reinforcement material can influence the thermal stability of metal matrices not to compromise the mechanical properties of nanocomposites. Since powder metallurgy is a promising method to produce Ni-CNT nanocomposites, this research aimed to investigate the effects of the microstructural evolution of nanocomposites during heat treatments. 

The heat treatments promoted nickel grain growth (and consequent hardness decrease), however, in the nanocomposites, the CNTs prevented this grain growth, helping to preserve the mechanical properties with the increase of temperature. The possible explanation is that the CNTs act as obstacles to the grain boundary movement when the temperature increases. The microstructures also showed smaller grains near the CNT agglomerates, confirming the grain boundaries pinning effect of the CNTs, hindering the grain growth mechanism. However, an inefficient CNT dispersion can compromise this effect, so this production step should be carefully analyzed, ensuring a uniform reinforcement dispersion in the matrix. 

The heat treatments did not induce the formation of an extensive reaction between the CNTs and the matrix and, therefore, it is not an essential factor for the thermal stability of the nanocomposites.

The CNTs also influence the density of the low-angle grain boundaries (LAGBs) and the recovery and recrystallization processes. For heat-treated nanocomposites, the LAGB density is higher than for the Ni matrix. The rearrangement and annihilation of dislocation during heat treatment are affected by the presence of CNTs as the dislocations are anchored in the clusters. This leads to nanocomposites having fewer recrystallized grains than the Ni matrix. For the effects of CNTs to cease to be effective, high temperatures (1100 °C) are needed for extended periods of time (e.g., 120 min). Heat treatments performed at 1100 °C induce graphitization which compromises the effects of reinforcement and interaction with dislocation and grain boundaries motion. Although, these conditions are very demanding and very different from those required in service. Therefore, it can be concluded that CNTs can actively improve the thermal stability of nanocomposites.

## Data Availability

Data are available upon request from the authors.
